# P-2154. A Multicenter Comparison of Recurrent Clostridioides difficile Infection Rates After Stem Cell Transplant Among Patients Treated with Oral Vancomycin or Fidaxomicin

**DOI:** 10.1093/ofid/ofaf695.2317

**Published:** 2026-01-11

**Authors:** Patrick T Zamba, Dilek Ince, Martha Carvour

**Affiliations:** University of Iowa Carver College of Medicine and College of Public Health, Iowa City, IA; University of Iowa Hospitals & Clinics, Iowa City, Iowa; University of Iowa Hospitals and Clinics, Iowa City, Iowa

## Abstract

**Background:**

Recurrent *Clostridioides difficile* infection (rCDI) can cause severe morbidity and mortality among adults who undergo a stem cell transplant (SCT). However, past research examining rCDI after SCT has relied largely on data from single healthcare organizations (HCOs) and small sample sizes (less than 500 adults). Larger studies across HCOs are needed to inform clinical practice guidelines.

**Methods:**

Using the TriNetX multicenter database, rCDI rates were examined among adults treated for CDI near the time of their SCT. Patients were eligible if they met the following criteria: (1) ≥ 18 years old; (2) SCT between January 1, 2015 and December 31, 2024; (3) any CDI diagnosis within 1 month before or after the SCT; and (4) any prescription for oral vancomycin and/or fidaxomicin within 1 month before or after the SCT. rCDI rates between 3-12 months after the SCT were estimated among patients who received oral vancomycin only, fidaxomicin only, or both (e.g., sequential use for recurrent or refractory CDI) during the CDI episode near the time of the SCT. rCDI rates for autologous and allogeneic SCT were also compared.

**Results:**

Of 1,733 patients with a CDI episode near the time of their SCT, 1,464 (84.5%) from 44 HCOs received oral vancomycin only, 122 (7.0%) from 24 HCOs received fidaxomicin only, and 147 (8.5%) from 25 HCOs received both agents during the 2-month window surrounding the SCT. rCDI was documented among 10.4% (n=152) of patients who received oral vancomycin only, compared to 8.2% (n=10) for those who received fidaxomicin only and 15.0% (n= 22) among patients who received both (p=0.15). rCDI was less frequent among patients who received autologous SCT (6.0%) compared to those who received allogeneic SCT (12.9%) (p< 0.0001).

**Conclusion:**

Overall, rCDI rates were lowest among patients who were only treated with fidaxomicin during the CDI episode near the time of the SCT and among patients who underwent autologous SCT. However, patients from 44 HCOs received oral vancomycin only while patients from 24 HCOs received fidaxomicin only; and reliable information about prophylactic use of oral vancomycin was not available. Further research is warranted to determine the role of system-level practice variations and patient-level factors on rCDI after a SCT.

**Disclosures:**

All Authors: No reported disclosures

